# Supplementary material for: Chromothripsis during telomere crisis is independent of NHEJ, and consistent with a replicative origin
Source: Genome Res. 2019 May;29(5):737–49. doi: 10.1101/gr.240705.118 (PMC6499312; doi:10.1101/gr.240705.118)
Supplement: Supplemental Material [file supp_gr.240705.118_Supplemental_file_1.zip › contigs/annotated_contigs/DB87/contig.2.DB87_length_272_mean_cov_3.86029411765.docx]

**DB87_length_272_mean_cov_3.86029411765**

```
TCCTGAAACAGGAGCTGATGAATTCCAGCGTGATGGAGGAGGGTTTGGATTGAACTCCAGGTGTCAATGAGGGACCTGGTTGGACACTT
 >chr18:10966068-10966168 - E=6e-49 p=2e-02
TCATTTTTGCA|ATTTTATAGATTCTATAAATTCGATTATAAATGCAAATTTTTAGAGTTGTGTTAATTTACAAAATTTTAACTTAAAC
             >chr18:66908133-66908305 - E=2e-92
CCTAAAGCCTTTCAATGACAATTCTAAATTCCTCTTTCATTTATTTAAGATTTTGTCTACATTTAGTTAAGAAATGCAAATACTTGCAG

ATTTGA
```
